# Correction: Solution-processed Cu_2_XSnS_4_ (X = Fe, Co, Ni) photo-electrochemical and thin film solar cells on vertically grown ZnO nanorod arrays

**DOI:** 10.1039/c8ra90072h

**Published:** 2018-09-03

**Authors:** Anima Ghosh, Dhirendra K. Chaudhary, Amrita Biswas, Rajalingam Thangavel, G. Udayabhanu

**Affiliations:** Solar Energy Research Laboratory, Department of Applied Physics, Indian Institute of Technology (Indian School of Mines) Dhanbad-826004 India rthangavel@gmail.com; Molecular Electronics Research Laboratory, Department of Physics, University of Allahabad Allahabad-211004 India; Department of Applied Chemistry, Indian Institute of Technology (Indian School of Mines) Dhanbad-826004 India; University of Michigan-Shanghai Jiao Tong University Joint Institute Shanghai-200240 China

## Abstract

Correction for ‘Solution-processed Cu_2_XSnS_4_ (X = Fe, Co, Ni) photo-electrochemical and thin film solar cells on vertically grown ZnO nanorod arrays’ by Anima Ghosh *et al.*, *RSC Adv.*, 2016, **6**, 115204–115212.

The authors regret that there were two errors in the original article. In the “Experimental details” section on page 115205, “1 M sodium sulfide at 70–80 °C for 24 h” should have read “0.5 M sodium sulfide at 70–80 °C for 24 h”. Additionally, Fig. 3 parts (b)–(d) were mistakenly reproduced from the authors’ previous publication (ref. 33 in the original article). The correct Fig. 3 is presented below.

**Fig. 3:**
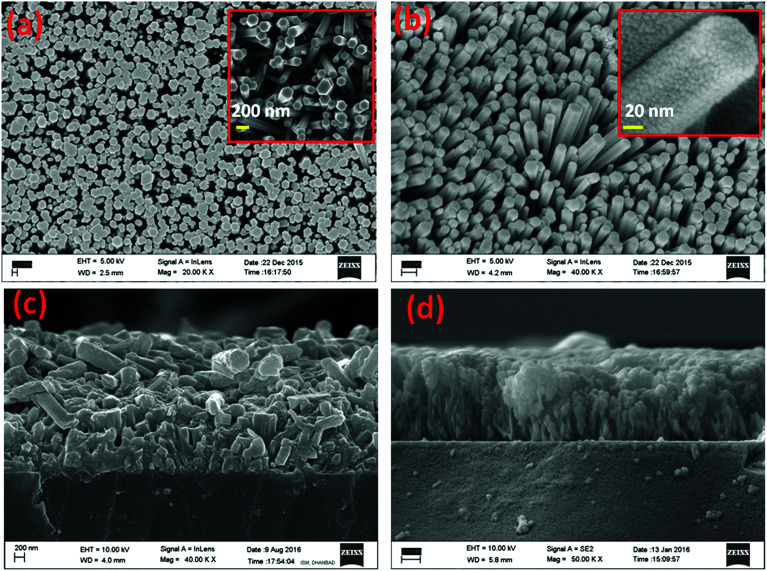
(a and b) FESEM images of ZnO nanorod arrays, ZnS sensitized ZnO nanorods; (c and d) cross-sectional images of ZnO nanorod arrays and ZnS sensitized ZnO nanorods. The inset in panel (a) shows ZnO nanorod arrays and the inset in panel (b) shows a magnified view.

The Royal Society of Chemistry apologises for these errors and any consequent inconvenience to authors and readers.

## Supplementary Material

